# The First Reported Case of VEXAS Syndrome in Lebanon: Efficacy of Azacitidine as a Therapeutic Option—Case Report

**DOI:** 10.1155/crh/3903470

**Published:** 2026-05-13

**Authors:** Roudy Issa, Bassem Akiki, Sophie Georgin-Lavialle, Olivier Kosmider, Danielle Hammond, Marcel Massoud, Vicky Najjar, Elissar Dagher

**Affiliations:** ^1^ Department of Internal Medicine and Clinical Immunology, Notre Dame des Secours University Hospital, Jbeil, Lebanon; ^2^ Department of Gastroenterology and Hepatology, Notre Dame des Secours University Hospital, Jbeil, Lebanon; ^3^ Department of Internal Medicine, Sorbonne University, AP-HP Tenon, Paris, France, sorbonne-universites.fr; ^4^ Department of Biological Hematology, Paris Cite University, AP-HP Cochin, Paris, France; ^5^ Department of Leukemia, The University of Texas MD Anderson Cancer Center, Houston, Texas, USA, mdanderson.org; ^6^ Department of Hematology/Oncology, Notre Dame des Secours University Hospital, Jbeil, Lebanon; ^7^ Department of Pathology, LAU Medical Center-Rizk Hospital, Beirut, Lebanon

**Keywords:** azacitidine, case report, myelodysplastic syndrome, VEXAS syndrome

## Abstract

**Background:**

Vacuoles, E1 enzyme, X‐linked, autoinflammatory, somatic (VEXAS) syndrome is a rare, adult‐onset autoinflammatory disease that has only been described since late 2020. Given the rarity of the disease and the absence of established treatment guidelines, management remains challenging and largely based on clinical experience and case reports.

**Case presentation:**

This paper reports the first documented case of VEXAS syndrome in Lebanon. A 73‐year‐old man presented with fever, severe asthenia, erythematous skin lesions, and anemia, initially diagnosed as giant cell arteritis. Following a diagnosis of VEXAS syndrome, he was treated with corticosteroids and methotrexate but developed refractory anemia and required erythropoietin therapy. Tocilizumab was introduced to manage inflammation, but the patient’s condition remained challenging due to corticosteroid dependence and myelodysplastic syndrome (MDS). Given the patient’s comorbidities and intermediate‐risk MDS, azacitidine was initiated as a therapeutic option. Despite initial neutropenia and infections, adjustments to the azacitidine regimen led to significant clinical and hematologic improvements. The patient achieved complete remission, became transfusion‐independent, and maintained stable hemoglobin levels.

**Conclusion:**

This case highlights the efficacy of azacitidine in managing VEXAS syndrome with MDS, particularly among patients ineligible for hematopoietic stem cell transplantation, offering a potential pathway to sustained remission and reduced corticosteroid dependence.

## 1. Introduction

Vacuoles, E1 enzyme, X‐linked, autoinflammatory, somatic (VEXAS) syndrome is an adult‐onset autoinflammatory disease first described by Beck and colleagues in December 2020 [1]. It is caused by somatic mutations in the *UBA1* gene located on the X chromosome and encodes ubiquitin‐activating enzyme 1, a key enzyme that initiates the protein ubiquitination process [2]. In VEXAS syndrome, these mutations are restricted to myeloid progenitors, leading to a unique clinical presentation that combines systemic inflammatory manifestations associated with hematological disorders [3–5].

VEXAS syndrome manifests with diverse inflammatory symptoms, such as fever, weight loss, chondritis, neutrophilic dermatosis, periocular edema, pulmonary involvement, thrombosis, and arthralgia. Hematological disorders typically include macrocytic anemia and thrombopenia [6–12]. The skin is the most frequently affected organ, with patients displaying various manifestations, such as neutrophilic dermatosis, purpuric papules, erythema nodosum, urticaria, erythematous papules, and livedo [1, 8, 9, 13].

Genetic diagnosis of VEXAS syndrome is established through *UBA1* gene sequencing [4, 8]. Given the rarity of the disease and the absence of established treatment guidelines, management remains challenging and is largely based on clinical experience and case reports. Therapeutic strategies often involve corticosteroids as first‐line therapy due to their efficacy in managing inflammatory symptoms. However, corticosteroid dependence is common, necessitating steroid‐sparing agents [14, 15]. Tocilizumab, an anti‐interleukin‐6 monoclonal antibody, has shown promise in reducing inflammation, though its use may be limited by specific side effects [16, 17]. Azacitidine, a hypomethylating agent, has emerged as a valuable treatment option, particularly for patients with concomitant MDS. Studies have demonstrated that azacitidine can induce significant clinical and hematologic responses in VEXAS patients, often allowing for the tapering of corticosteroids [18] and achieving complete remission after treatment cessation [19–21]. The mechanism by which azacitidine exerts its effects in VEXAS syndrome may involve both anti‐inflammatory and immunomodulatory actions [22, 23]. Lastly, allogeneic hematopoietic stem cell transplantation (HSCT) remains the only curative treatment option and should be considered early in the disease course. Nevertheless, its non‐negligible morbidity and mortality risks require careful patient selection, considering factors such as age and comorbidities [24].

## 2. Case Presentation

**FIGURE 1:**
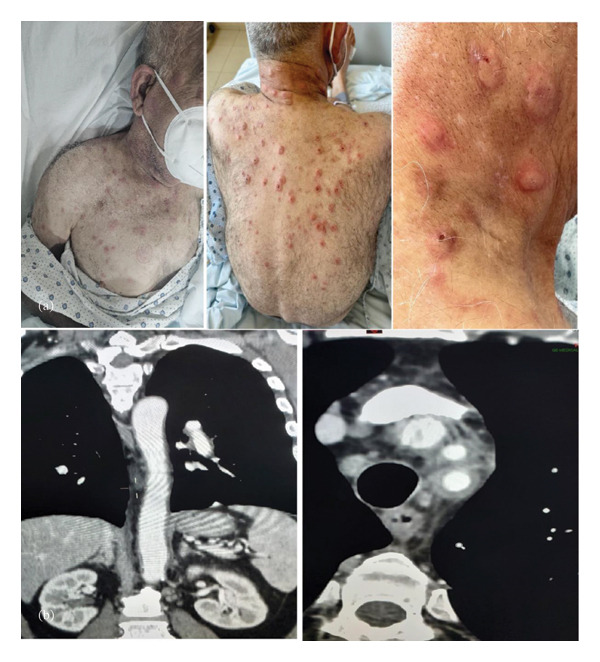
(a). Annular and papular erythematous skin lesions on the neck, thorax, trunk, and limbs. A biopsy of the cutaneous lesions demonstrated the presence of neutrophilic dermatosis. (b). A total body scan showing a circumferential wall thickening of the thoracic and abdominal aorta in favor of large vessel vasculitis.

A 73‐year‐old man presented in September 2018 with a fever (39°C–40°C) lasting more than a week, accompanied by severe asthenia, anorexia, and skin lesions (Figure 1(a)). His medical history included well‐controlled Type 1 diabetes, arterial hypertension, dyslipidemia, severe coronary artery disease (with stents in the left anterior descending artery and lesions in the circumflex and right coronary arteries), and a subtotal colectomy for colonic polyposis. The patient received initial empirical antibiotic treatment, but extensive testing ruled out infection. Blood tests revealed a severe inflammatory syndrome (CRP > 400 mg/L [reference range: < 5 mg/L], ESR > 100 mm/hr [reference range: 35–37 mm/hr]), normocytic anemia (Hb = 11 g/dL [reference range: 13.5–17.5 g/dL]), and mild hyponatremia (Na^+^ = 131 mEq/L [reference range: 135–145 mEq/L]). A whole‐body scan showed circumferential wall thickening of the thoracic aorta and pulmonary artery, consistent with large‐vessel vasculitis (Figure 1(b)). Based on these findings, giant cell arteritis without cranial involvement was suspected, and high‐dose corticosteroids led to marked clinical and biological improvement. Bone marrow biopsy revealed mildly hypercellular marrow with granulocytic hyperplasia.

Following clinical improvement, the patient was discharged on tapering doses of corticosteroids; however, his course was complicated by corticosteroid dependence, with symptoms such as recurrent febrile episodes, asthenia, and inflammatory flare‐ups requiring dose increases. Skin biopsies revealed neutrophilic dermatosis. In March 2021, methotrexate (MTX) at 15 mg/week was introduced as a steroid‐sparing agent, successfully reducing the prednisone dose to 7 mg/day. By September 2021, worsening anemia necessitated erythropoietin therapy, leading to improved hemoglobin levels. However, in June 2022, anemia became refractory to treatment, and MTX was discontinued due to suspected bone marrow suppression.

Treatment options were discussed in a multidisciplinary meeting, and corticosteroids were used as first‐line therapy.

**FIGURE 2:**
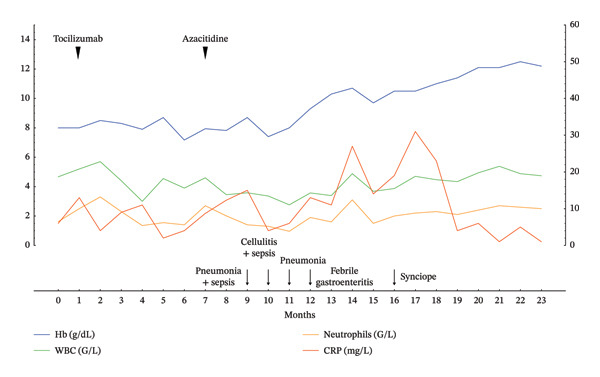
Variations in hemogram levels and CRP during tocilizumab and azacitidine treatment, along with associated complications (January 2023–December 2024). All the blood tests were done before any transfusion of packed red blood cells (if required). Hemoglobin level in g/dl. WBC count in G/L. Absolute neutrophil count in G/L. CRP level in mg/L.

Given patient comorbidities, tocilizumab was selected for its efficacy in large‐vessel vasculitis [25]. Initiated in January 2023, tocilizumab improved inflammatory symptoms, allowing prednisone tapering to 6 mg/day (Figure 2). However, skin lesions flared after MTX discontinuation.

**FIGURE 3:**
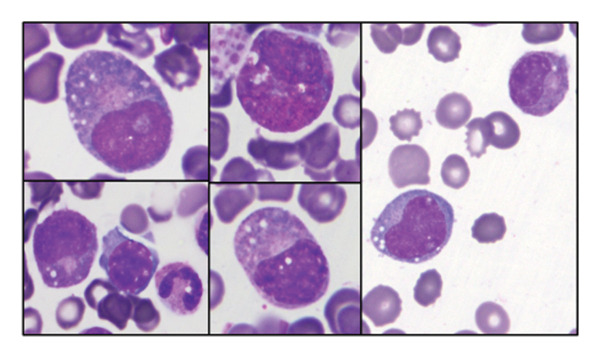
Bone marrow aspirate smear with granulocyte precursors in various stages showing cytoplasmic vacuoles (100x).

In April 2023, a bone marrow biopsy and aspirate smear demonstrated mildly hypercellular marrow with dysplastic features, cytoplasmic vacuolization, and a chromosome 7 deletion at band q22 (del7q) (Figure 3). Based on the Revised International Prognostic Scoring System (IPSS‐R), the patient was classified as having an intermediate‐risk myelodysplastic syndrome (MDS).

As *UBA1* genetic testing is not available in Lebanon, physicians and laboratories often send blood or bone‐marrow samples abroad. In the present case, peripheral blood was sent overseas in April 2023 to Hôpital Cochin in Paris, France, for genetic testing. The turn‐around time was around 3 weeks, which was in line with previously published data [26]. Testing was performed free of charge due to the collaboration with Notre Dame des Secours Hospital.

**FIGURE 4:**
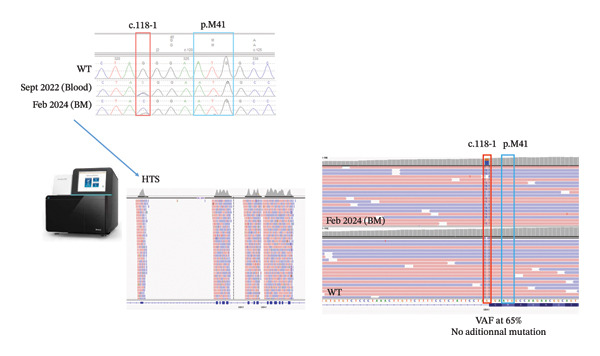
Sanger sequencing of a peripheral blood sample in September 2022 and Next Generation Sequencing (NGS) of a bone marrow sample in February 2024 showing a mutation in the *UBA1* gene type splice 1, directly impairing the translational start site at p.Met41 and causing VEXAS syndrome.

Sanger sequencing confirmed a splice‐site mutation (c.118‐1G > C) in the *UBA1* gene, with a variant allele frequency of 65% in bone marrow, leading to the diagnosis of VEXAS syndrome (Figure 4).

After the VEXAS diagnosis, the treatment strategy changed, and azacitidine was initiated in July 2023. HSCT was not considered a viable option due to patient age and comorbidities. Ruxolitinib was also excluded due to cardiovascular risks. Since opportunistic infections are commonly reported in VEXAS syndrome [27], the patient was receiving valacyclovir 500 mg/day as prophylaxis, and during episodes of neutropenia he was treated with levofloxacin 500 mg/day.

During the initial phase of azacitidine treatment, which was obtained by the patient out‐of‐pocket from France, hemoglobin levels stabilized at 7.5 g/dL with transfusions every 3 weeks, although neutropenia persisted (Figure 2). Despite severe clinically suspected infections requiring hospitalization, including pneumonia, cellulitis, and bacterial gastroenteritis [28, 29], all cultures were negative, and no microorganisms were detected. The dose of azacitidine was adjusted to 100 mg/day for 5 days every 3 weeks to improve tolerance and maintain therapeutic efficacy. The adjusted azacitidine regimen, combined with prednisone, stabilized the patient’s condition. After eight cycles of azacitidine, neutrophilic dermatosis improved significantly, although skin lesions remained dependent on timely administration of the treatment. The prednisone dose was tapered to 6 mg/day. By February 2024, a new myelogram revealed only mild dysplastic features and a reduced percentage of precursors showing cytoplasmic vacuoles. Given the patient’s history of multiple infectious complications, persistent anemia, and mild neutropenia, and following consultation with international experts, azacitidine was tapered to 50 mg/day for six consecutive days every 3 weeks. As a result, the patient became transfusion‐independent, with stable hemoglobin levels around 12 g/dL and normal white blood cell (reference range: 4.0–11.0 g/L) and platelet counts (reference range: 150–450 × 10^9^/L) (Figure 2). The patient achieved a complete clinical and hematologic remission of VEXAS syndrome with no inflammatory flare‐ups, despite skin lesions remaining sensitive to delays in azacitidine administration. The time to reach clinical and hematologic remission (from the initiation of azacitidine, including subsequent dose adjustments) was 13 months (approximately 17 cycles).

## 3. Discussion and Conclusions

This case report presents the first documented instance of VEXAS syndrome in Lebanon. At the time of the diagnosis, VEXAS was a newly recognized disease entity, not yet known in Lebanon, making it particularly challenging to diagnose and manage. The patient’s complex presentation, which included large‐vessel vasculitis, cutaneous lesions, MDS with hematologic abnormalities, severe inflammatory syndrome, and high corticosteroid dependence, further contributed to the diagnostic and therapeutic challenges.

As a cosmopolitan disease, VEXAS syndrome can affect patients worldwide, necessitating a global perspective on its diagnosis and management. Given the rarity and complexity of this syndrome, effective treatment strategies remain a subject of ongoing research.

In the absence of standardized evidence‐based therapeutic guidelines for VEXAS syndrome, several therapeutic options have been proposed. The most recent guidelines from the American College of Rheumatology Guidance Statement for the Diagnosis and Management of VEXAS, developed by the International VEXAS Working Group Expert Panel, recommend HSCT in case of transfusion‐dependent anemia and MDS as the first‐line therapy or the use of azacitidine if HSCT is not viable. In the present case, the patient presented with both inflammatory and hematologic manifestations, including transfusion‐dependent anemia and MDS. Thus, he received azacitidine, as he was not a candidate for HSCT [30, 31]. Pharmacological options encompass systemic corticosteroids and steroid‐sparing agents such as conventional DMARDs, interleukin‐1 receptor antagonists, interleukin‐6 receptor antagonists (tocilizumab), tumor necrosis factor‐alpha inhibitors, Janus kinase inhibitors, and hypomethylating agents (azacitidine) [14].

The present case illustrates the effectiveness of azacitidine in achieving complete clinical and hematologic remission in a patient with VEXAS syndrome and intermediate‐risk MDS. Initially, the patient required transfusions every 3 weeks, but after adjusting the azacitidine regimen, he became transfusion‐independent with stable hemoglobin levels. This outcome aligns with reports from other studies where azacitidine treatment resulted in sustained remission and reduced corticosteroid dependence [18, 19, 21, 32]. However, azacitidine treatment can be complicated by severe infections and cytopenia, necessitating careful dose adjustments and monitoring [20, 28, 33]. The ability to taper azacitidine doses while maintaining remission, as observed in our case, suggests that personalized treatment strategies may be effective in managing VEXAS syndrome.

Despite promising results, no predictive factors for response to azacitidine have been identified in patients with VEXAS syndrome and myelodysplasia. Notably, in many cases, hematological and clinical remission has been accompanied by molecular and genetic remission for several months, even after discontinuation of azacitidine, suggesting a direct impact on the clonal evolution of mutated *UBA1* cells, with either complete clearance or significant reduction of the *UBA1* mutant clone in the bone marrow [18, 20, 21].

The patient expressed satisfaction with the treatment received and reported noticeable improvement in symptoms following the intervention. They appreciated clear communication from the medical team and felt supported throughout the care process.

In conclusion, this case supports the use of azacitidine as a therapeutic option for VEXAS syndrome, particularly in patients with MDS or those who are not candidates for HSCT. Further research is needed to fully elucidate the mechanisms of action of azacitidine in VEXAS syndrome and establish evidence‐based treatment guidelines for this complex condition.

**Nomenclature**
HSCT: Hematopoietic stem cell transplantation
IPSS‐R: International Prognostic Scoring System
MDS: Myelodysplastic syndrome
MTX: Methotrexate
VEXAS: Vacuoles, E1 enzyme, X‐linked, autoinflammatory, somatic

## Author Contributions

Roudy Issa: Conceptualization and writing–review and editing.

Bassem Akiki: conceptualization and review and editing.

Sophie Georgin‐Lavialle: interpretation and review and editing.

Olivier Kosmider: interpretation and review and editing.

Danielle Hammond: interpretation and review and editing.

Marcel Massoud: interpretation and review and editing.

Vicky Najjar: interpretation and review and editing.

Elissar Dagher: conceptualization, review and editing, and supervision.

Dr. Elissar Dagher had full access to all of the data in this study and takes complete responsibility for the integrity of the data and the accuracy of the data analysis.

## Funding

This research did not receive any specific grant from funding agencies in the public, commercial, or not‐for‐profit sectors.

## Disclosure

All authors have read and approved the final version of the manuscript and agree to its submission to the journal.

## Ethics Statement

This study was conducted following the ethical standards of the Declaration of Helsinki. It was approved by CHU‐Notre Dame des Secours Institutional Review Board (date of IRB approval 6/2/2025; ethical approval number CR: 1/2025).

## Consent

Written informed consent was obtained from the patient for the publication of this case report, including relevant clinical details and accompanying images.

## Conflicts of Interest

The authors declare no conflicts of interest.

## Data Availability

Data are available from the corresponding author upon request.
